# Full recovery after prolonged resuscitation in a pediatric patient due to fulminant myocarditis: a case report with three-year follow-up

**DOI:** 10.1186/s12887-022-03158-9

**Published:** 2022-02-16

**Authors:** Yanping Gu, Peng Xue, Hong-Lin Chen, Guojin Zou, Yongcheng Ni, Lin Li, Lijuan Lu, Hao Chen, Aibin Zheng

**Affiliations:** Affiliated Changzhou Children’s Hospital of Nantong University, No.468, middle Yanling Road, Tianning District, Changzhou City, Jiangsu province People’s Republic of China

**Keywords:** Pediatric patients, Fulminant myocarditis, Prolonged resuscitation, Full recovery, Case report

## Abstract

**Background:**

Fulminant myocarditis (FM) is a common life-threatening disease in pediatric patients, which can result in sudden cardiac arrest (CA). Whether prolonged cardiopulmonary resuscitation (CPR) is beneficial to FM induced CA is unknown.

**Case presentation:**

We reported the case of an 8-year-old child with FM. At 14:49 of the day after admission, the ECG monitoring indicated ventricular flutter. The patient was immediately given continuous external cardiac compression. Electric cardioversion (energy 30J) and electric defibrillation (energy 50 J, 100 J, 100 J) were given. Continuous chest compression was conducted until extracorporeal membrane oxygenation (ECMO) successfully placed at 19:30 P.M. The total duration of CPR was 291 min. Nine days later, the ECMO was removed; and 29 days later, the patient was discharged from hospital. In the three years of follow-up, the boy showed a full recovery without neurological sequela. At present, his daily activities have returned to normal and his academic performance at school is excellent.

**Conclusions:**

Prolonged CPR can be used in FM induced in-hospital CA in pediatric patients, especially during preparation for ECMO after the failure of standard resuscitation measures.

## Background

Fulminant myocarditis (FM) is a life-threatening disease in pediatric patients, which is a diffuse acute inflammatory disease of the myocardium. The disease progresses rapidly and often leads to death. The three main factors that may contribute to or result in FM include viral infections, autoimmune disease and drug toxicity [1]. Most patients presented with acute congestive heart failure, severe arrhythmias, Adams-Stokes syndrome, and cardiogenic shock [1, 2]. Sudden cardiac arrest (CA) also may occur in patients with FM [3].

Cardiopulmonary resuscitation (CPR) is the standard life-saving rescue method for cardiac arrest. But the effect is not particularly satisfactory. A recently review analyzed 15,000 children who received CPR for in-hospital CA found that as many as 80% to 90% survive the event, but most patients do not survive to hospital discharge [4]. Clinicians have attempted to improve the prognosis by prolonging CPR (> 30 min). According to R.A Berg's report on outcomes of CPR in PICU's, 66% of children younger than 18 years survived to hospital discharge after 1–3 min of CPR compared with 28% after more than 30 min; among survivors, 90% had a favorable neurologic outcome after 1–3 min of CPR compared with 89% after more than 30 min CPR [5]. In another in hospital pediatric CA study, Matos reported a survival probability of 41% for CPR lasting 1 to 15 min and 12% for CPR duration of more than 35 min. Among survivors, favorable neurological outcome occurred in 70% who underwent < 15 min of CPR and 60% who received CPR for > 35 min [6]. Many patients survived after prolonged CPR. But the maximal duration of CPR for CA is still unknown.

In this study, we reported for the first time a child presenting with in-hospital CA due to FM who survived after receiving prolonged CPR (291 min) followed by extracorporeal membrane oxygenation (ECMO) treatment. At present, after 3 years of follow-up, the boy has shown a full recovery without neurological sequela. This case report provides new thought for the maximum duration of CPR for FM induced in hospital CA in pediatric patients.

## Case presentation

On September 20, 2018, an 8 years and 9 months old boy came to Affiliated Changzhou Children's Hospital of Nantong University for emergency treatment. The boy presented with abdominal pain and fever for 2 days. The initial diagnosis was gastroenteritis. On arrival to our hospital, he was listlessness with frequent vomiting. His heart rate (HR) was 104 bpm with blood pressure (BP) of 83/53 mmHg. Physical examination showed that the abdomen was flat and soft without tenderness and rebound pain. Laboratory examination indicated that troponin was positive, aspartate transferase was 164 U/L, creatine kinase was 1082.5 U/L, and creatine kinase isoenzyme was 97 U/L. The admission diagnosis was FM. The patient was then transferred to the intensive care unit (ICU), where the disease progressed rapidly and cardiogenic shock occurred. His BP remained unstable despite intravenous dopamine, with a minimum reading of 57/30 mmHg. At 9:25 on September 21, his electrocardiogram (ECG) showed a ventricular fibrillation rhythm which resolved spontaneously before defibrillation was initiated. He was then intubated and place on mechanical ventilation.

**Fig. 1:**
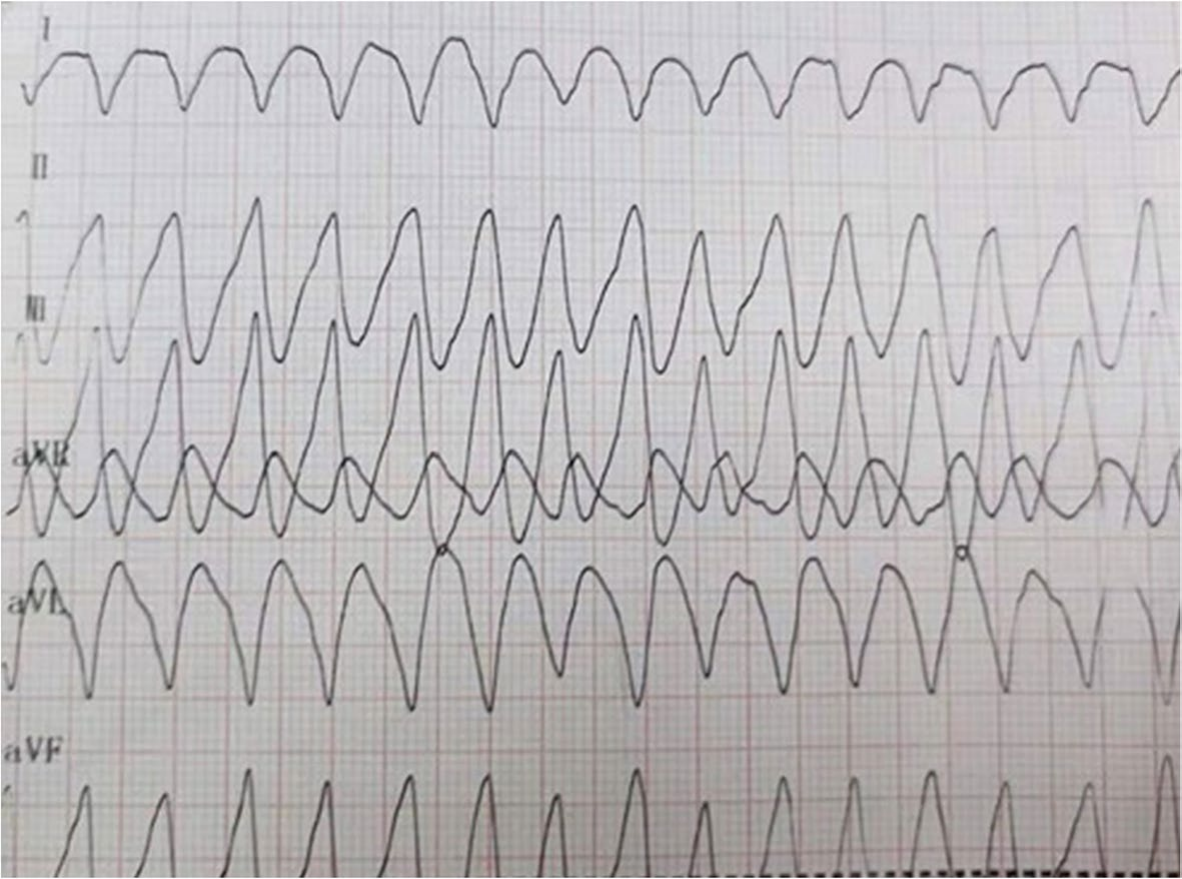
During hospitalization, the ECG monitoring indicated ventricular flutter, at 14:49 P.M. on September 21, 2018

At 14:49 on September 21, the ECG monitoring indicated ventricular flutter (Fig. 1), the invasive arterial BP reading was undetected and no heart sounds were heard. Continuous external cardiac compression was immediately initiated, IV epinephrine (1:10,000) administered, and electric cardioversion (30 J) given, followed by electrical defibrillation (50 J, 100 J, 100 J). Despite these measures the ECG waveform did not change significantly. During CPR no recordable BP was measured when cardiac compression was stopped. Continuous chest compression was continued until ECMO was successfully placed at 19:30 on September 21. From 14:49 to 19:30, the total duration of CPR was 291 min. A medical team of more than 30 people participated in the manual compressions and rotated every 2 min. For staff who developed fatigue prior to 2 min, the compressions were taken over by another staff member. No rib fractures occurred in the patient during compressions. The patient's condition stabilized after ECMO, which was maintained until removal on September 29 after which the patient was transferred to the cardiology unit. The patient was successfully discharged home on October 19.

**Fig. 2:**
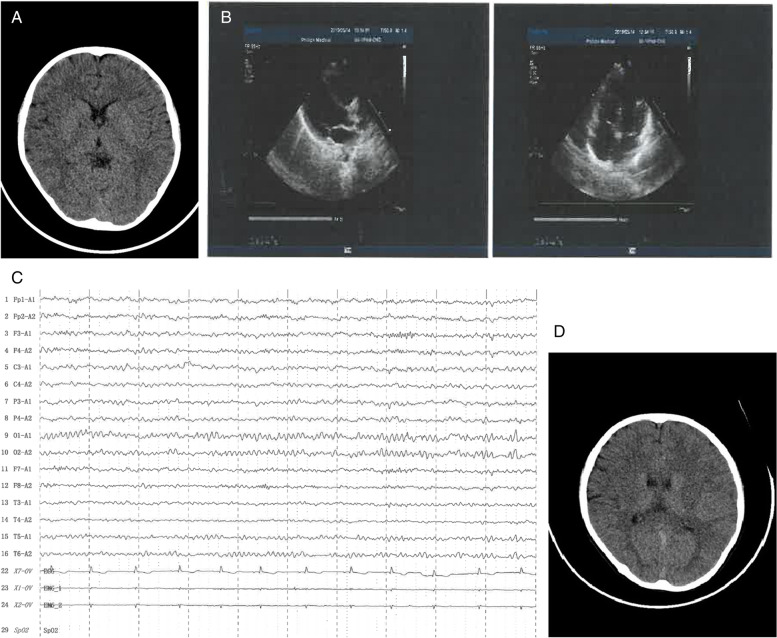
During follow-up **A** CT scan showed slightly high density shadow in the left occipitoparietal lobe on October 23, 2018; **B** Color doppler echocardiography showed that the intracardiac structure was normal, left ventricular systolic function was normal, and EF was 72% on May 14, 2019; **C** EEG showed no clinical events on September 26, 2020; **D** head CT scan showed normal image on July 9, 2021

After discharge, the patient was followed up for 3 years. On October 23, 2018, the child developed a facial twitch and mouth salivation. Head CT scan showed a slightly high density shadow in the left occipitoparietal lobe (Fig. 2A). Oxcarbazepine was given orally. On November 9, 2018, ECG showed sinus rhythm, color doppler ultrasound indicated normal heart function despite slight thickening of the ventricular wall. On May 14, 2019, ECG showed sinus rhythm, and ST elevation in leads II and AVF of about 0.05 MV. Color doppler echocardiography showed that the systolic function of left heart was normal, ejection fractions (EF) was 72% (Fig. 2B). On September 12, 2020, ECG showed sinus rhythm, color doppler echocardiography showed that the left ventricular systolic function was normal, EF was 69%, with mild tricuspid regurgitation. Intelligence test (WISC-IV) showed that the total intelligence quotient (IQ) was 105, speech comprehension index was 113, perceptual reasoning index was 106, working memory index was 88, and processing speed index was 104. In June 2020, oxcarbazepine was stopped, and there were no further convulsions. Electroencephalograms (EEGs) on September 26 2020 and September 26 2021 showed no clinical events (Fig. 2C). On July 9, 2021, head CT scan showed a normal image (Fig. 2D). At present, his daily activities have returned to normal and his academic performance at school is excellent.

## Discussion and conclusion

In this case report, we present a child with FM induced CA, who fully recovered without neurological sequela after prolonged CPR. The total duration of CPR was 291 min. We speculate that the following factors may have contributed to his successful recovery from prolonged CPR.

First, this is a case of FM induced CA in a pediatric patient. In the ICU, the FM disease progressed rapidly and cardiogenic shock occurred. The patient had hypotension one hour before CPR, and the average arterial pressure was maintained at about 40 mmHg for more than 1.5 h. Our patient survived without significant neurological sequelae during the 3 years of follow-up, suggesting that an average arterial pressure of 40 mmHg may be sufficient to maintain cardiac and cerebrovascular perfusion in pediatric patients. Sugamura et al. also reported a 20-year-old female who survived FM after 56 h of non-responsive CA. This patient also developed cardiogenic shock refractory to pharmacological intervention, and was able to resume a normal life with intraaortic balloon pumping [7]. These 2 cases support the assertion that prolonged CPR should be considered in FM induced CA in younger patients.

Second, the CA of the patient occurred in the ICU. This was an in-hospital CA and not an out-of-hospital event. Early effective CPR is closely related to the prognosis of CA. Times to initiation of CPR greater than 2 min were associated with a survival of 14.7% as compared with 17.1% if CPR was begun in 2 min or less (adjusted odds ratio, 0.68 [0.54 to 0.87]) [8]. The survival rate was only 4% after 6 min resuscitation, and it was almost impossible to succeed after 10 min resuscitation [9]. For this child, CPR started as soon as CA occurred, which ensured adequate blood perfusion to vital organs, particularly the heart and brain. We therefore speculate that under the premise of in-hospital CA and immediate CPR, prolonged CPR can be attempted for FM-induced CA.

Third, ECMO life support was carried out for this pediatric patient. Asaumi et al. found ECMO to be a highly effective form of haemodynamic support for FM patients; once patients recovered from inflammatory myocardial damage, the subsequent clinical outcome was favourable, similar to that observed in patients with acute non-FM [10]. For FM induced CA, Li et al. present the case of a 9-year-old girl with FM complicated by CA, she received effective CPR followed by ECMO. The girl recovered with intact cardiac and neurocognitive functions after continued ECMO treatment for 221 h [11]. So, we are therefore of the opinion that CPR duration can be extended in FM induced CA, which buy time for ECMO to be commenced.

In conclusion, FM is a life-threatening disease that can occur in children. When patients develop in-hospital CA, prolonged CPR can be beneficial particularly when preparing to start ECMO following failure of standard resuscitation measures. We hope that our findings benefit other cardiologist when managing pediatric patients with FM induced in-hospital CA.

## Data Availability

All data generated or analyzed during this study are included in this published article.

## Abbreviations

BP: Blood pressure
CA: Cardiac arrest
CPR: Cardiopulmonary resuscitation
ECG: Electrocardiogram
ECMO: Extracorporeal membrane oxygenation
EF: Ejection fractions
FM: Fulminant myocarditis
HR: Heart rate
